# Electronic Publishing and the Journals of the American Chemical Society

**DOI:** 10.6028/jres.101.037

**Published:** 1996

**Authors:** Jeffrey D. Spring, Lorrin R. Garson

**Affiliations:** American Chemical Society, 1155 16th Street, NW, Washington, DC 20036

**Keywords:** CD-ROM, Crystallographic Information File (CIF), electronic advertising, e-mail, World Wide Web

## Abstract

The American Chemical Society is developing a number of initiatives that implement emerging electronic technologies in order to provide a broad range of products and services to members and subscribers. Examples of products currently available, or under development, for access via the World Wide Web include supporting information for journals, electronic ads, color graphics and entire journals. Other activities employ e-mail, CD-ROMs, and softcopy text.

## 1. Introduction

The American Chemical Society (ACS) publishes or co-publishes 23 journals in various fields of chemistry and chemical engineering as well as several magazines and approximately 40 books per year. In 1995, approximately 117,000 pages of original, scientific research were published in these journals. In addition, 80,000 pages of Supporting Information (previously called Supplementary Material) were supplied by authors. With this much material to present to subscribers, it is imperative that the ACS pursues alternative avenues of distribution from the traditional printed journals.

The ACS has seen trends in the papers presented in recent years that are both encouraging and daunting. There has been an annual increase of 10 % in the number of manuscripts being submitted to the Society. Also, the average length of a manuscript has been increasing. This trend is due to an increase in instrumentation data being included and, possibly, an increase in verbosity on the part of authors brought on by the ease of writing and “repackaging” articles through the use of computers. At the same time as the length and number of manuscripts is increasing, the cost of producing the journals has also increased. The cost for producing journals is proportional to the number of pages printed. Composition costs currently run $150 to $200 per printed page.

As a consequence of these trends, ACS has a strong incentive to seek a reduction in the length of printed manuscripts in order to keep subscription prices down. There is also a need to maintain clear readability of the articles by assuring that key points the author is trying to put forth are not lost in the verbiage. Also, while traditional print journals will continue to serve an essential role for the foreseeable future, alternative means of information distribution are coming to the forefront which must be utilized where appropriate. For example, electronic versions of manuscripts have distinct advantages such as full text searching and the ability to provide true digital delivery.

## 2. Electronic Publishing Activities

The American Chemical Society has a number of electronic products and services under development or already available to subscribers. What follows is a review of the most important projects currently underway.

### 2.1 Activities on the World Wide Web

Several exciting products and services have recently been developed to take advantage of the burgeoning use of the World Wide Web (WWW) as a means of information dissemination. The ACS Publications Division Web server (http://pubs.acs.org) currently has links to nine different products and services offered by the Division. These include “Hot Articles” from ACS periodicals, supporting information from the journals, and advertisements from companies advertising in *Chemical & Engineering News, Chemcyclopedia*, and *Today’s Chemist at Work* through ACS’s “Advertised Product Information Network.”

#### 2.1.1 Hot Articles

Text and graphics from selected articles of general interest are being placed on-line under the “Hot Articles” option. Participating magazines include *Chemical & Engineering News, Analytical Chemistry, CHEMTECH, Today’s Chemist at Work, Chemical Health & Safety*, and *Environmental Science & Technology*. When possible, the material is loaded on the publication date so it is often available on the Web prior to the printed version arriving in subscribers’ mailboxes.

#### 2.1.2 Supporting Information for Journals

Since July 1995, electronic supporting information has been available to current journal subscribers via the Internet using either WWW or gopher. The supporting information on-line for the *Journal of the American Chemical Society* (*JACS*) goes back to January 1993 and to January 1995 for all other journals. Most of the material provided to date has been posted in PDF format and requires the use of Abobe’s Acrobat Reader to read. However, some material is available as computer programs, word processing files, or in standardized formats such as the CIF (Crystallographic Information File) for crystallographic data.

In general, the supporting information is only available to subscribers of the journals the material is from. However any CIF material from *JACS, Inorganic Chemistry, Organometallics*, or *Chemistry of Materials* is available to subscribers to any one of these journals. A large percentage of the supporting information for these journals is crystallographic material, providing a great opportunity to increase the use of the CIF standard. While supporting information loaded in PDF format can only be downloaded in page images, CIF material is ASCII in nature meaning it can be used to verify crystal structures or downloaded into data files.

The initial project design for the acceptance of CIF material called for the CIFs to be e-mailed from the authors to an address at ACS and then automatically forwarded, via e-mail, onto the appropriate editorial office. If a reviewer wanted to see the CIF during the review process, it would have to be e-mailed from the editorial office. However, when a file was forwarded by the UNIX e-mailer at ACS to the editorial offices it was split into multiple files by the Eudora e-mail software used in most of the offices. This required the editorial staff to piece the file back together.

A second approach has since been initiated. The authors still e-mail the files to ACS, but they are not forwarded to the editorial office. Instead, they are loaded into a “non-public” address on the Publications Division Web server. Reviewers who want to download the CIF must point their browser to the server and enter a password to access the CIF. This process will greatly reduce the clerical involvement of the editorial staff. Once the paper associated with a CIF is accepted, the CIF will be moved to the public Supporting Information site on the Publications Web page.

#### 2.1.3 Electronic Advertising on the WWW

Advertising is a significant source of revenue for *Analytical Chemistry, Chemical & Engineering News, Environmental Science & Technology*, and *Today’s Chemist at Work*. Starting in the spring of 1995, the ACS launched a project that offers advertisers in these periodicals the opportunity to place ads on the Web. It is expected that providing ads in this format will broaden the market rather than compete with the traditional print audience. In 1995, the service was provided without extra charge to invited advertisers of *Chemical & Engineering News* and *Today’s Chemist at Work*. For 1996, there is a charge for the services. These services are available 24 hours a day world wide and provide the users with the opportunity to pinpoint the specific products of interest. Users receive the desired additional information almost instantly, rather than waiting weeks after filling out reader service cards.

The electronic advertising effort has two components that utilize different types of electronic technology. The Advertised Product Information Network places ads on the World Wide Web. Currently, there are 15 participating companies with a total of 39 ads. Most of the material is text and graphics, but there is one movie. The material is currently organized by company, but near term plans include providing a search engine so users can find information on a desired topic. Plans also call for the addition of a standard response form for users to request additional product information directly from the advertisers. Response to this service, both from advertisers and the users of the Web site, has been enthusiastic.

The second service, the Electronic Reader Service, is designed to provide users with additional information about a product they saw in a print ad in an ACS publication. In the associated print ad, there is an e-mail address to which interested persons are to send e-mail requesting additional information. Each ad also contains a unique code that is to be referenced in the e-mail to determine the text file for transmission back to the requester. So far, response to this service has been weak.

### 2.2 Color Graphics

ACS is also testing the feasibility of making color graphics available to subscribers on the Web. The cost of providing color material over the Web is a fraction of the current cost to print color in journals. It might be feasible to print black and white graphics in the hardbound journals with a reference to a WWW site where the color version would be available.

### 2.3 CD-ROMS

*The Journal of Physical Chemistry* and the *Journal of the American Chemical Society* have been available in a CD-ROM format since January 1994. Supporting information for a given journal is also included on that journal’s CD. To date, most of the supporting information has been provided in TIFF format, although true digital formats will be placed on the CDs as they become available. The articles are fully searchable and the CDs are mastered every two months.

### 2.4 Softcopy of Accepted Manuscripts

While manuscripts submitted for review are still required to be in hardcopy format, since January 1994, manuscripts that have been accepted for publication in ACS journals have been composed from disks submitted by authors. From January 1994 to August 1995, the percentage of manuscripts being accepted in softcopy format has increased from 4 % to 73 %. Currently, text, tables and simple, in-line equations are being converted. Graphics are still handled in the traditional photographic process. Well over 90 % of the files submitted on disk are either WordPerfect or Microsoft Word, and these files are passed through a filter into FrameMaker. Editing changes are made in FrameMaker and then the files are converted to Xyvision for photocomposition.

### 2.5 JACS and CJACS Plus on STN

Two of the first electronic products from ACS were CJACS and CJACS Plus. CJACS is a searchable, full text file that goes back to 1984. CJACS Plus contains page images for all journals in TIFF format from 1992. The CJACS file resides on STN International where it is accessible world-wide via dial-up modems or the Internet. Three nodes exist for STN: one each in Columbus, Ohio; Karlsruhe, Germany; and Tokyo, Japan. The CJACS file is searchable using standard Boolean statements against a fielded file. Proximity operations are allowed.

While the CJACS file offers customers an outstanding research and delivery tool, it is inadequate as a document delivery mechanism. The CJACS file does not contain graphics, tables, or mathematics which are essential to the understanding of chemical communications. To remedy this deficiency, complete papers in TIFF format were added and are called the CJACS-Plus file. This permits customers to download, view and print articles retrieved by the sophisticated search capability offered by CJACS. These documents may also be delivered to customers by FAX.

### 2.6 Journal of Physical Chemistry on WWW

In 1996, the 100th anniversary of *The Journal of Physical Chemistry*, the journal will be made available on the World Wide Web to subscribers. The material will be composed as a compound document of searchable text linked to the associated graphics. This will be the first ACS journal with a complete version on the Internet.

## 3. The Future

As with all endeavors associated with the rapidly changing world of electronic communications, trying to predict what activities the American Chemical Society will be involved in requires as much creative and fanciful thinking as technological know-how. More journals and directories will surely provide some or all their content via the Internet. There will be increased interactive opportunities for readers to send in “letters to the editor” or obtain additional information on topics from editorial policies to product information via electronic mail options. As bandwidth to the users increases, there will be more services incorporating sound, video, and additional use of graphics. With whatever electronic tools that come into play, the ACS will continue to strive to provide subscribers with the best original research in chemistry.

